# Chronic non-healing ulcer reveals pyoderma gangrenosum: A case report

**DOI:** 10.1177/2050313X251350372

**Published:** 2025-06-16

**Authors:** Orhan Yilmaz, Philip Doiron, Robyn Evans

**Affiliations:** 1 College of Medicine, University of Saskatchewan, Saskatoon, SK, Canada; 2 Division of Dermatology, Department of Medicine, Faculty of Medicine, University of Toronto School of Medicine, ON, Canada; 3 Wound Care Centre, Women’s College Hospital, Toronto, ON, Canada

**Keywords:** pyoderma gangrenosum, granulomatous dermatitis, chronic ulcer, topical corticosteroids

## Abstract

Pyoderma gangrenosum is a rare, ulcerative skin condition with complex diagnostic and therapeutic challenges, particularly in cases without associated systemic conditions. We present the case of a 71-year-old female with a chronic, non-healing ulcer, initially unresponsive to conventional topical treatments. After a thorough evaluation and biopsy, a diagnosis of pyoderma gangrenosum was made. Treatment efforts included both intralesional and topical therapies, with varying responses observed. This case highlights the challenges in diagnosing and managing pyoderma gangrenosum, emphasizing the need for individualized care and a multidisciplinary approach to treat chronic, non-healing ulcers.

## Introduction

Pyoderma gangrenosum (PG) is a rare inflammatory dermatosis often presenting as painful, rapidly progressing ulcerations.^1^ It typically occurs with systemic diseases such as inflammatory bowel disease, rheumatoid arthritis, or hematologic malignancies. However, idiopathic cases are also reported.^2,3^ Diagnosing PG is challenging, as it is essentially a clinical diagnosis of exclusion and can mimic various infectious, autoimmune, and neoplastic conditions.^4^ Histopathologic findings in PG, while helpful, are nonspecific and often reveal neutrophilic infiltration and suppurative inflammation, which require a comprehensive clinical and laboratory evaluation to confirm.^4^ Treatment traditionally involves systemic immunosuppressive therapies, but localized therapies can be effective for limited disease.^4^ Here, we discuss a case of a patient whose ulcer was challenging to diagnose. The initial trial of intralesional corticosteroid was ineffective and may have worsened the ulcer, raising concerns about the diagnosis of PG and the risk of infection. Further biopsies and a review of the biopsies indicated PG as a possibility. This case underscores the importance of individualized treatment and a multidisciplinary approach to PG, especially in idiopathic and localized disease.

## Case presentation

**Figure 1.**
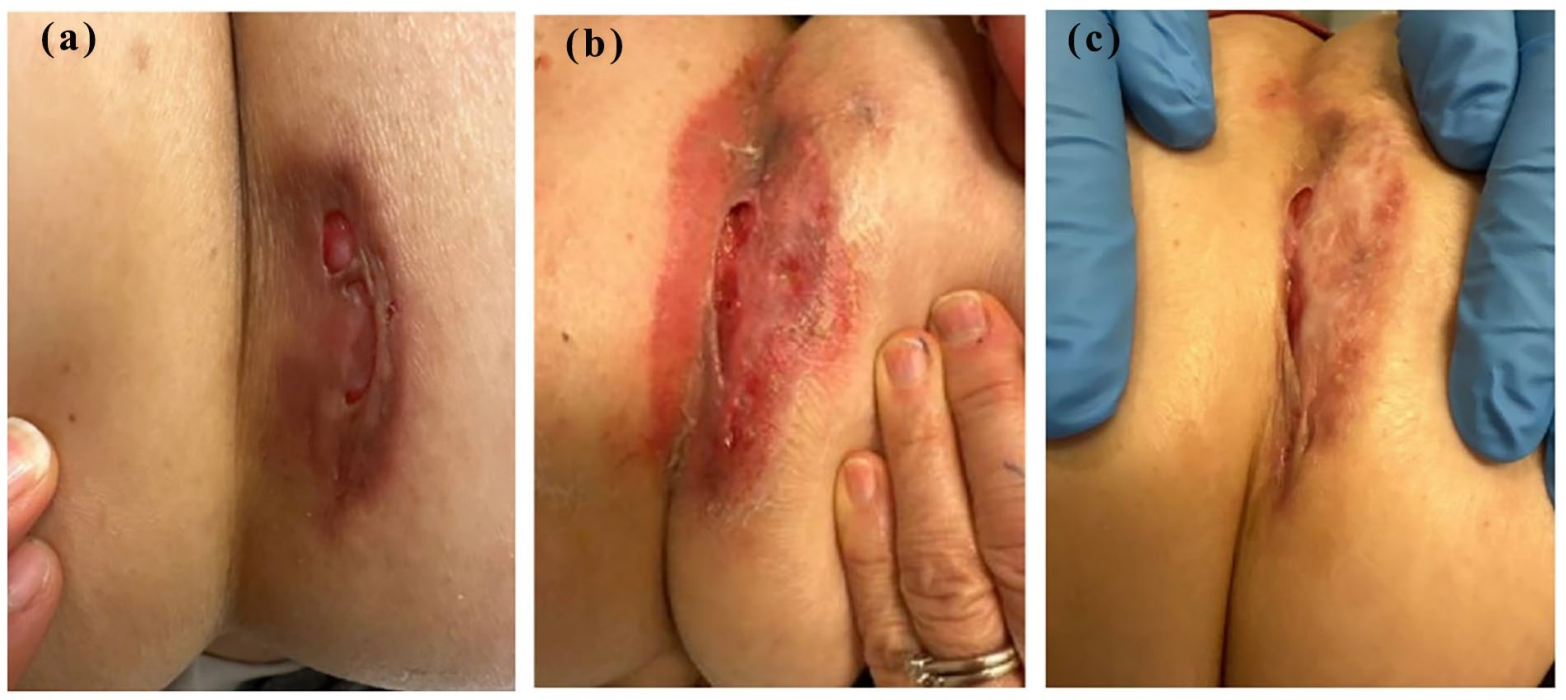
(a) Seventy-one-year-old woman with a chronic, non-healing ulcer located on her right flank at the time of presentation, (b) 8 months later, after the ulcer worsened, and (c) a partial resolution after treatment with topical clobetasol 0.05% cream for 2 months.

A 71-year-old female with a history of diabetes and thyroid disease presented to the clinic with a single 6 × 2 cm chronic, non-healing ulcer located on her right flank (Figure 1(a)). The ulcer had, at times, appeared to improve and almost heal. She reported that she thought it was related to the elasticity of her uniform rubbing in the area. She had tried various topical treatments, including silver sulfadiazine, antimicrobial textile dressings, and paraffin gauze dressings, with limited improvement. The lesion was associated with erythema, granulation tissue, and occasional serous discharge. Although generally not painful, the lesion exhibited mild tenderness to palpation and increased discomfort when pressure was applied from lying or sitting on the affected side.

The patient’s clinical evaluation and laboratory investigations aimed to identify an underlying cause. There were no constitutional symptoms, nor any symptoms concerning for inflammatory bowel disease or inflammatory arthritis. The initial two biopsies indicated granulomatous dermatitis. Multiple differential diagnoses were considered, including sarcoidosis, deep fungal infections, atypical mycobacterial infections, and autoimmune conditions commonly associated with ulcerative skin manifestations. Comprehensive infectious testing, including multiple sets of fresh tissue cultures for bacteria, fungi, and mycobacteria, and serologic tests (HIV, hepatitis panels, syphilis serology, and TB interferon gamma release assay) returned negative results. The autoimmune workup, including anti-nuclear antibodies (ANA), anti-neutrophil cytoplasm antibodies (ANCA), rheumatoid factor (RF), and anti-cyclic citrullinated peptide (anti-CCP), was negative, effectively ruling out autoimmune conditions commonly associated with similar skin presentations. Workup for malignancy was negative. With infectious causes ruled out, an intralesional corticosteroid (triamcinolone acetonide 5 mg/mL) was administered. At the follow-up appointment 1 month later, the ulcer had seemed to worsen, with increased erythema and lesion expansion (Figure 1(b)). Due to a remote history of travel, a tropical disease consult was initiated, ruling out leishmaniasis. A clear diagnosis was not evident during this extensive workup. A request was made for a second pathology opinion on the two earlier biopsies. The re-read of the slides indicated epithelial ulceration with neutrophilic abscess and multinucleated giant cells. Differential diagnosis included infection or PG in the appropriate clinical context.

Following this new biopsy report, topical clobetasol 0.05% cream was reintroduced and applied with each dressing change, gradually improving the lesion. Follow-up evaluations demonstrated progressive healing (Figure 1(c)), supporting the diagnosis of PG and reducing the likelihood of alternative inflammatory causes.

## Discussion

This case illustrates the diagnostic complexity and clinical challenges of managing chronic non-healing ulcers like PG. PG is a rare, inflammatory neutrophilic dermatosis, often associated with underlying systemic disorders, including inflammatory bowel disease, hematologic malignancies, and autoimmune diseases.^2^

Histopathological findings in PG typically include neutrophilic infiltration and suppurative inflammation, which were evident in this case.^4^ However, these findings are not pathognomonic, making the diagnosis predominantly clinical and one of exclusion. The negative infectious and autoimmune workups supported the PG diagnosis, as did the positive response to high-potency topical corticosteroids, often the first-line treatment for localized PG lesions. Topical clobetasol has shown efficacy in controlling inflammation and promoting healing, as observed in this patient’s case. Despite initial worsening with intralesional corticosteroid, topical clobetasol provided effective, localized symptom management.

Prompt recognition and appropriate treatment of PG are essential to avoid progression and complications, which may include extensive tissue destruction and scarring. Regular follow-up and wound care were integral to managing the lesion and monitoring for signs of recurrence or systemic involvement. This case underscores the importance of a multidisciplinary approach, including dermatology and wound care, in managing PG and the potential benefit of steroid therapy in controlling localized PG without the need for systemic immunosuppression.

## Conclusion

This case highlights the diagnostic challenges and treatment considerations for PG. Although PG often requires systemic immunosuppressive therapy, high-potency topical corticosteroids, like clobetasol, can provide effective local treatment and help avoid systemic immunosuppression in select cases. The patient’s response to clobetasol following exacerbation with intralesional steroid underscores the necessity of individualized treatment plans in PG and the importance of close follow-up in managing refractory chronic ulcers. A multidisciplinary approach, incorporating dermatology and wound care expertise, is essential to optimizing patient outcomes in complex cases of PG.
